# Obesity and chronic kidney disease: A population-based study among South Koreans

**DOI:** 10.1371/journal.pone.0193559

**Published:** 2018-02-28

**Authors:** Lorraine S. Evangelista, Won-Kyung Cho, Youngmee Kim

**Affiliations:** 1 Sue & Bill Gross School of Nursing, University of California, Irvine, California, United States of America; 2 International Health Care Center, Asan Medical Center, University of Ulsan College of Medicine, Seoul, Republic of Korea; 3 Red Cross College of Nursing, Chung-Ang University, Seoul, Republic of Korea; Istituto Di Ricerche Farmacologiche Mario Negri, ITALY

## Abstract

Obesity and chronic kidney disease (CKD) are major global health problems. There are very little data concerning the prevalence and its associated factors of obesity in non-dialyzed patients who have different stages of CKD. Therefore, in this study, we examined the prevalence of obesity and its associated factors according to the stages of CKD. We used nationwide representative data from the Korean National Health and Nutrition Examination Survey, which was conducted over a 7-year period from 2008 to 2014 by the Korea Centers for Disease Control and Prevention. The results indicated that: (1) general obesity and abdominal obesity were more prevalent in patients with CKD compared to those without CKD; (2) the prevalence of general obesity and abdominal obesity was highest in stage 2 CKD; (3) stages 3a and 3b were the factors associated with general obesity, and stage 3a was significantly associated with abdominal obesity; (4) the association between general obesity/abdominal obesity and CKD disappeared in people with advanced stage 4/5 CKD; and (5) the presence of comorbidities contributed to the development of both general obesity and abdominal obesity. The findings of this study might support the idea that weight loss is a good potential intervention for the prevention of disease progression in moderate CKD (stage 3), but not severe CKD (stage 4/5).

## Introduction

Obesity is a worldwide health threat. Socio-economic burden and the prevalence of obesity worldwide has increased substantially over the last 3 decades [1]. Obesity is a well-established risk factor for cardiovascular diseases and type-II diabetes, which together predispose people to developing chronic kidney disease (CKD) [2]. Numerous population-based studies have established a significant association between obesity and the development and progression of CKD [3–9]. Given that the deleterious effects of obesity on CKD have been well documented, it is interesting that obesity is consistently reported to lower the mortality rate in patients with advanced CKD and end stage kidney disease (ESKD) [7, 10–12]. Therefore, we believe that more questions regarding the relationship between obesity and CKD still remain unanswered. In this study, we have hypothesized the following: if obesity is a crucial contributor to the development of CKD, the prevalence of obesity should increase with an increase in the stage of CKD, and if there is a significant relationship between CKD and obesity, CKD itself might act as an independent factor that is associated with the development of obesity. To test these hypotheses, we examined the prevalence of obesity based on the 5 different stages of CKD. We then investigated the factors that showed an independent association with obesity using a particular CKD stage as an independent variable. This study was performed using a representative nationwide survey to test our hypotheses in Koreans who represent an ethnically homogenous population.

## Materials and methods

### Study design, data sources, and participants

The present study is a secondary data analysis of the Korean National Health and Nutrition Examination Survey (KNHANES) which was performed over a 7-year period from 2008 to 2014 by the Korea Centers for Disease Control and Prevention (KCDC). The KNHANES is an ongoing, nationwide, population-based, cross-sectional, multistage, stratified, and clustered probability sampling survey with the aim of minimizing sampling bias and ensuring that the sample represents the South Korean population [13]. The survey, which is conducted annually, targets non-institutionalized civilians and consists of a self-reported questionnaire/interview (e.g., psychosocial behaviors and health-related symptoms), laboratory tests and a nutritional survey (e.g., dietary habits).

**Fig 1:**
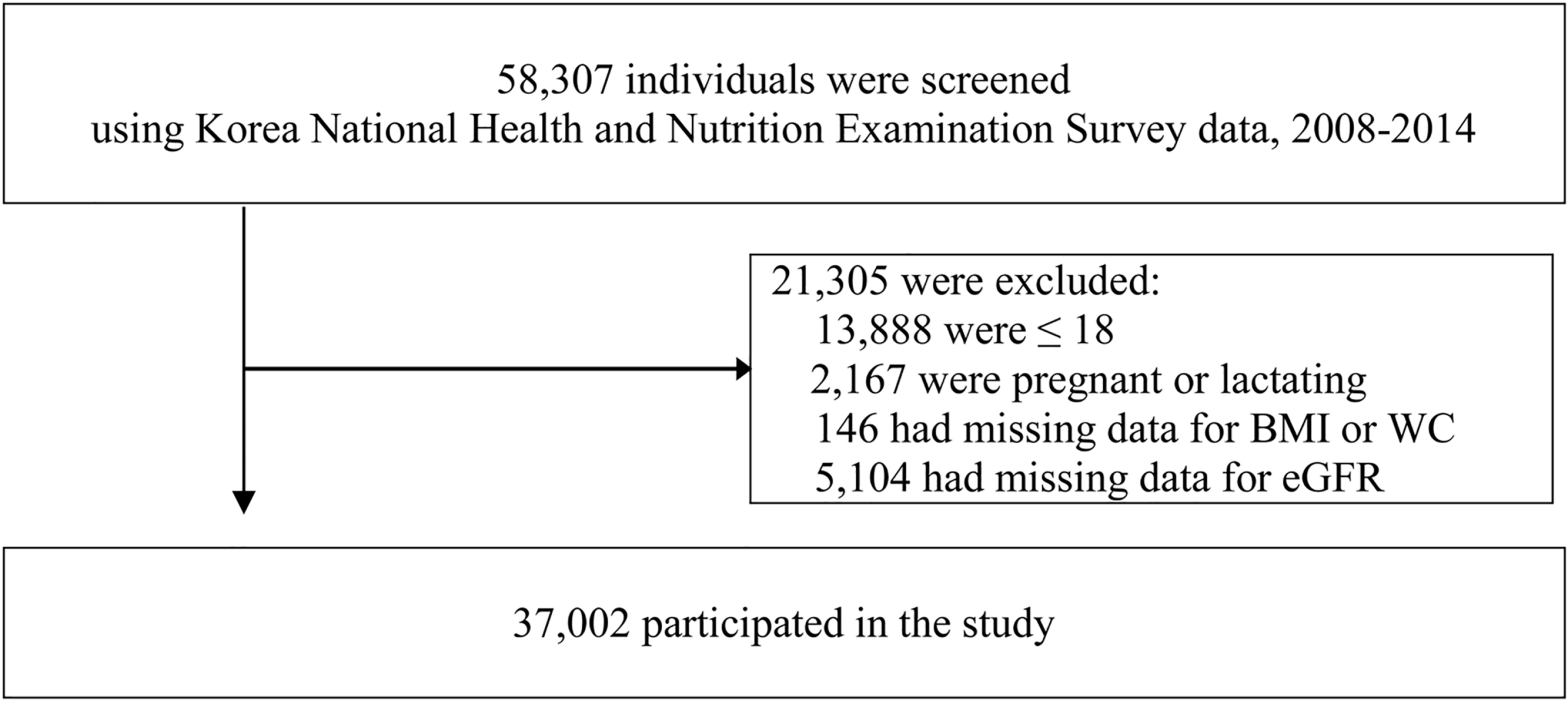
Flow diagram showing the study participants.

In the present study, we included adults aged ≥ 19 years and excluded: (1) those who were pregnant or lactating, (2) those who had missing data for body mass index (BMI) or waist circumference (WC), and (3) those who had missing data for estimated glomerular filtration rate (eGFR). A total of 58,307 individuals were screened, and 37,002 people were included in the study according to the inclusion and exclusion criteria. Fig 1 demonstrates the selection process and the number of study participants.

General obesity was defined as BMI ≥ 25 kg/m2 based on the cut-off value for Asians [14]. Abdominal obesity was defined as waist circumference (WC) ≥ 90 cm for men and ≥ 85 cm for women based on the recommended cut-off values for Koreans

### Ethics statement

The KNHANES survey is reviewed and approved by the Institutional Review Board of the Korea Centers for Disease Control and Prevention on an annual basis (Approval numbers: 2008-04EXP-01-C, 2009-01CON-03-2C, 2010-02CON-21-C, 2011-02CON-06-C, 2012-01EXP-01-2C, 2013-07CON-03-4C, 2013-12EXP-03-5C). Each study participant provided written informed consent before the survey. Data were downloaded from the official website of the KNHANES (http://knhanes.cdc.go.kr/). These data are open to the public after completing a designated registration process for access [13]. This study used only de-identified existing data.

### Measurements and definitions of major clinical and general characteristics

#### General obesity and abdominal obesity

Body mass index (BMI) was calculated as weight in kg divided by height in m2. General obesity was defined as BMI ≥ 25 kg/m2 based on the cut-off value for Asians [14]. Abdominal obesity was defined as waist circumference (WC) ≥ 90 cm for men and ≥ 85 cm for women based on the recommended cut-off values for Koreans [14].

#### Estimated glomerular filtration rate (eGFR)

Serum creatinine level was measured using the kinetic Jaffe method with an ADVIA 1650 Analyzer (Siemens, Tarrytown, NY, USA) and/or a Hitachi Automatic Analyzer 7600 (Hitachi, Tokyo, Japan) in 2008–2012 and the rate-blanked and compensated Jaffe method with a COBAS 8000 C70 (Roche, Mannheim, Germany) in 2013–2014. Blood samples were obtained after an overnight fast [13].

The eGFR was calculated using the Modification of Diet in Renal Disease Study equation [15]. Chronic kidney disease was categorized into five stages according to the Kidney Disease: Improving Global Outcomes (KDIGO) staging system [16]; CKD stage 3 was further refined into stages 3a and 3b [17]. Specifically, the CKD stages were defined as follows: CKD stage 1 = eGFR ≥ 90 mL/min/1.73 m2 and proteinuria determined by dipstick urine test ≥ 1+; CKD stage 2 = eGFR 60–89 mL/min/1.73 m2 and proteinuria; CKD stage 3a = eGFR 45–59 mL/min/1.73 m2; CKD stage 3b = eGFR 30–44 mL/min/per 1.73 m2; and CKD stage 4/5 = eGFR < 30 mL/min/1.73 m2.

#### Sociodemographic, health-related, and clinical characteristics

Sociodemographic characteristics of the participants included age, sex, educational level, residential area, household income quartiles and employment status. Health behaviors included alcohol drinking and smoking habits, and mental health included perceived health status assessed by self-reported questionnaires.

Alcohol drinking was defined as the consumption of more than seven glasses of alcoholic drinks per occasion, more than two times per week. Current smoking was defined as both currently smoking and having smoked ≥ 100 cigarettes in a participant’s lifetime. Regular exercise was defined as exercising at a moderate level of intensity (≥ 30 min per session) more than five times per week or a rigorous level of intensity (≥ 20 min per session) more than three times per week. Perceived health status was defined as the level of overall health (i.e., very good/good, fair, or poor/very poor) perceived by the person. Health-related quality of life (HRQoL) was measured using the EuroQoL, which uses both a health-status descriptive system (EQ-5D) and a visual analogue scale (EQ-VAS) ranging from 0 to 100: 0 signifies the worst HRQoL while 100 indicates the best HRQoL [18]. Hypertension (HTN) was defined as systolic blood pressure (SBP) ≥ 140 mmHg, diastolic blood pressure (DBP) ≥ 90 mmHg, or use of any antihypertensive medications. Diabetes mellitus (DM) was defined as a fasting blood glucose (FBG) ≥ 126 mg/dL, use of antidiabetic medications, or a physician’s diagnosis of any type of DM. Anemia was defined as hemoglobin (Hb) level < 13 g/dL in men and < 12 g/dL in women. Other comorbidities such as cancer, angina pectoris, acute myocardial infarction, stroke, chronic obstructive pulmonary disease or asthma were based on the physician’s diagnosis. Cancer was defined as a medical history of any type of cancer.

#### Statistical analysis

Data were analyzed using SAS version 9.4 (SAS Institute, Inc., Cary, NC, USA). A p-value < 0.05 was considered statistically significant. All data are presented as means ± standard error (SE) for continuous variables or as proportions (±SE) for categorical variables. Analysis of variance and chi-square tests were performed to evaluate the differences in continuous and categorical variables between the groups. The prevalence of general obesity and abdominal obesity according to eGFR was analyzed as a proportion (±SE). Sociodemographic and clinical characteristics, including mental health and comorbidities, were also examined according to eGFR.

Hierarchical multiple logistic regression analyses were conducted to explore the associations between general obesity, eGFR and all outcomes of interest. The same approach was followed to explore the associations between abdominal obesity, eGFR, and these outcomes. In the analyses, Model 1 was unadjusted, and Model 2 was adjusted for the variables in Model 1 and sociodemographic factors (age, sex, education, employment status). Model 3 was adjusted for the variables in Model 2 and health-related characteristics (sleep duration, smoking, alcohol drinking), and Model 4 was adjusted for the variables in Model 3 and comorbidities (cancer, ischemic heart disease, chronic obstructive pulmonary disease). Finally, Model 5 was adjusted for the variables in Model 4, SBP, DBP, and laboratory measurements (fasting blood glucose, total cholesterol, hemoglobin). The EuroQoL (VAS) was assessed only in 2010–2012, and ferritin was measured only in 2008–2012. Therefore, these factors were omitted from the adjustments. The results were reported using adjusted odd ratios (ORs) and their 95% confidence intervals (CIs). A 95% CI that did not span 1.0 was considered statistically significant.

## Results

### Prevalence of general obesity and abdominal obesity according to eGFR

The prevalence rates of general obesity and abdominal obesity in the study participants were 32.5% and 24%, respectively (Table 1). The prevalence rate of CKD in the study cohort was 5.0%, and the prevalence rates of the different stages of CKD according to eGFR levels were 0.3% (Stage 1), 0.8% (Stage 2), 3.4% (Stage 3a), 0.6% (Stage 3b) and 0.2% (Stage 4/5). The prevalence rates of general obesity were 37.7%, 53.8%, 43.6%, 45.5%, and 37.8% in patients with CKD stage 1, 2, 3a, 3b and 4/5, while the prevalence of general obesity in the non-CKD group was 32.0%. In addition, abdominal obesity occurred in 29.7%, 45.2%, 40.6%, 40.2%, and 30.7% of patients with CKD stages 1, 2, 3a, 3b, and 4/5, while the rate of abdominal obesity in non-CKD patients was 23.4%. Thus, the prevalence of general obesity and abdominal obesity was greater in the CKD group than in the non-CKD group, with the prevalence rates of general obesity and abdominal obesity being highest in patients with CKD stage 2 (p < .001).

**Table 1:** Prevalence of obesity and abdominal obesity according to eGFR stages.

	Total	Non-CKD	Stage1	Stage2	Stage3a	Stage3b	Stage4/5	P
Obesity	32.5%	32.0%	37.7%	53.8%	43.6%	45.5%	37.8%	< .001
Abdominal obesity	24.0%	23.4%	29.7%	45.2%	40.6%	40.2%	30.7%	< .001

P-values by Chi-square test

### Sociodemographic, health-related, and clinical characteristics according to eGFR levels

Table 2 presents the sociodemographic characteristics of the study participants. Age, sex, education level, living area, living with a spouse or not and employment status were significantly different among the groups. In particular, mean age tended to increase with the higher CKD stages up through stage 3b (p < .001). The unemployment rate was highest in patients with CKD stage 4/5 (p < .001).

**Table 2:** Sociodemographic characteristics of the 37,002 study participants.

Characteristics	Total (N = 37,002)	Non-CKD (n = 35,135)	CKD Stage 1 (n = 107)	CKD Stage 2 (n = 229)	CKD Stage 3a (n = 1,251)	CKD Stage 3b (n = 208)	CKD Stage 4/5 (n = 72)	*p*
Age	45.1±0.15	44.4±0.2	40.6±1.5	54.4±1.1	66.9±0.5	69.1±1.0	59.5±2.4	< .001
≤39	39.5 (0.5)	40.7(0.5)	50.3(5.7)	19.2(3.4)	1.3(0.45)	0.7(0.7)	11.8(5.7)	< .001
40–49	22.6(0.4)	23.1(0.4)	23.3(5.2)	18.7(3.4)	6.4(1.1)	5.9(2.2)	9.6(4.6)	
50–59	18.9(0.3)	18.8(0.3)	15.3(4.5)	24.8(3.4)	19.9(1.5)	16.1(3.7)	25.5(6.0)	
60–69	10.9(0.2)	10.4(0.2)	7.3(2.3)	19.3(2.7)	23.5(1.4)	23.0(3.5)	19.6(5.8)	
≥70	8.2(0.20)	6.9(0.18)	3.8(1.6)	18.1(2.8)	48.9(1.8)	54.4(4.2)	33.4(6.2)	
Sex								.009
Male	54.7(0.28)	54.6(0.29)	61.0(5.5)	68.4(3.5)	54.1(1.7)	55.1(4.2)	53.0(7.1)	
Female	45.3(0.28)	45.4(0.29)	39.0(5.5)	31.6(3.5)	45.9(1.7)	44.9(4.2)	47.0(7.1)	
Education								< .001
Elementary to Middle school	27.6(0.4)	26.5(0.4)	24.4(4.9)	42.0(3.9)	61.6(1.8)	70.9(4.0)	49.8(7.6)	
High school to University	72.4(0.4)	73.5(0.4)	75.6(4.9)	58.0(3.9)	38.4(1.8)	29.1(4.0)	50.2(7.6)	
Residential area, rural	18.8(1.0)	18.6(1.0)	15.4(3.8)	20.6(3.7)	24.8(1.9)	23.3(3.4)	17.4(5.2)	.001
Living without spouse	28.6(0.4)	28.6(0.5)	32.5(5.6)	18.7(3.4)	28.0(1.6)	38.6(4.0)	35.1(6.6)	.011
Household income								.288
Lower to Lower middle	50.5(0.6)	50.5(0.6)	53.8(5.7)	54.6(4.1)	46.9(1.9)	55.7(4.2)	52.4(7.1)	
Middle upper to Upper	49.5(0.6)	49.5(0.6)	46.2(5.7)	45.4(4.1)	53.1(1.9)	44.3(4.2)	47.6(7.1)	
Currently Unemployed	34.3%(0.37)	33.4(0.4)	42.7(6.0)	45.7(3.9)	59.0(1.8)	75.2(3.7)	75.9(6.4)	< .001

***Note*:** 1) Values are sample n, weighted mean ± standard error (SE) or sample n, weighted percentage (SE) unless otherwise indicated; p-value by analysis of variance (ANNOVA) or chi-square test as appropriate; 2) See Methods for the definitions of CKD stages.

Table 3 displays the differences in various clinical characteristics according to CKD stages. The mean SBP and presence of comorbidities such as DM or anemia were highest in patients with stage 4/5 CKD (p < .001). The rates of poorer perceived health status and suicidal ideation were also highest in patients with CKD stage 4/5 (p < .001). Among people with CKD, mean BMI was highest in patients with CKD stage 2 and lowest in those with CKD stage 4/5 (p < .001). Mean WC was highest in patients with stage 2 CKD and lowest in those with stage 1 CKD (*p* < .001). The serum levels of total and LDL cholesterols were highest in stage 2 CKD patients (p < .001). Current smoking and alcohol drinking are more common in patients with CKD stage 1 and 2 among the CKD group.

**Table 3:** Health-related and clinical characteristics of the study participants.

Characteristics	Total (N = 37,002)	Non-CKD (n = 5,135)	CKD Stage 1 (n = 107)	CKD Stage 2 (n = 229)	CKD Stage 3a (n = 1,251)	CKD Stage 3b (n = 208)	CKD Stage 4/5 (n = 72)	*p*
***Health-related Factors***								
Sleep duration (hr.)	6.8±0.01	6.8±0.01	7.1±0.2	7.0±0.1	6.7±0.1	6.5±0.1	7.2±0.3	.020
Current smoking	27.7(0.3)	27.9(0.3)	34.0(5.7)	30.7(3.8)	15.6(1.3)	20.8(3.6)	19.7(5.5)	< .001
Alcohol drinking	60.9(0.4)	61.5(0.4)	69.0(5.5)	60.6(3.9)	38.8(1.7)	36.9(4.2)	37.3(7.4)	< .001
Regular exercise	10.7(0.3)	10.8(0.3)	9.2(3.4)	7.4(1.9)	8.3(1.0)	10.3(2.6)	7.4(3.5)	.190
Perceived health status								< .001
Very good/ Good	37.1(0.4)	37.5(0.4)	26.0(5.1)	32.8(3.9)	27.7(1.7)	20.4(3.4)	11.1(4.5)	
Fair	45.3(0.4)	45.6(0.4)	46.4(5.9)	38.8(3.9)	38.4(1.7)	30.2(4.0)	17.5(5.1)	
Poor/ Very poor	17.6(0.3)	16.9(0.3)	27.6(4.8)	28.4(3.6)	34.0(1.7)	49.4(4.4)	71.3(6.33)	
EQ-5D	0.95±0.0	0.95±0.0	0.94±0.01	0.92±0.01	0.88±0.01	0.82±0.02	0.81±0.03	< .001
EuroQoL: VAS	74.6±0.2	74.8±0.2	72.5±2.3	71.4±1.6	69.7±0.8	62.1±2.3	61.6±2.4	< .001
Depressive symptom (Yes)	13.0(0.2)	12.9(0.2)	18.3(4.8)	17.1(3.1)	14.3(1.3)	22.1(3.7)	18.5(5.8)	.012
Perceived psychological stress	27.3(0.3)	27.5(0.3)	26.7(5.1)	28.8(3.7)	20.5(1.4)	18.9(3.2)	20.7(6.0)	.001
Suicidal ideation	13.2(0.3)	12.9(0.3)	9.5(3.5)	14.1(2.8)	19.4(1.5)	26.1(3.9)	34.6(7.5)	< .001
***Comorbidity***								
Cancer	2.4(0.1)	2.3(0.1)	-	4.0(1.5)	5.2(0.7)	4.6(1.6)	1.9(1.5)	Not estim-able
Ischemic heart disease	1.7(0.1)	1.4(0.1)	4.0(2.5)	4.7(1.6)	7.9(0.9)	14.0(3.0)	8.3(3.2)	< .001
Stroke	1.3(0.1)	1.1(0.1)	0.8(0.8)	5.3(1.6)	7.0(0.8)	11.0(2.8)	10.6(4.2)	< .001
Hypertension	24.4(0.3)	22.8(0.3)	37.2(5.8)	61.6(3.9)	67.0(1.7)	76.3(3.6)	87.9(3.6)	< .001
DM	8.7(0.2)	7.7(0.2)	33.2(5.6)	36.0(4.0)	30.1(1.7)	45.8(4.3)	54.8(7.8)	< .001
Anemia	7.1(0.2)	6.5(0.2)	6.7(2.6)	10.3(2.2)	18.0(1.2)	40.7(4.2)	86.8(4.6)	< .001
COPD or Asthma	3.1(0.1)	3.0(0.1)	1.0(0.8)	3.9(1.3)	5.9(0.8)	4.9(1.5)	4.0(2.5)	< .001
***Exam/Lab Tests***								
SBP (mmHg)	116.8±0.1	116.4±0.1	122.1±2.0	131.8±1.7	126.9±0.7	128.0±2.0	134.1±3.1	< .001
DBP (mmHg)	75.7±0.1	75.7±0.1	81.1±1.3	82.7±1.1	75.5±0.4	73.4±1.1	76.5±2.2	< .001
BMI (kg/ m^2^)	23.7±0.0	23.7±0.0	23.7±0.5	25.3±0.3	24.6±0.1	24.6±0.3	23.5±0.5	< .001
WC (cm)	81.3±0.1	81.1±0.1	81.8±1.3	87.3±0.9	85.5±0.3	85.9±0.8	82.9±1.5	< .001
Cr (mg/dL)	0.9±0.0	0.8±0.0	0.8±0.0	1.0±0.0	1.2±0.0	1.6±0.02	3.5±0.3	< .001
Total-C (mg/dL)	187.4±0.3	187.3±0.3	199.7±4.7	204.8±3.7	189.0±1.3	181.2±3.9	177.0±5.1	< .001
LDL-C	113.3±0.5	113.5±0.5	97.8±7.3	123.7±6.6	110.6±2.5	98.5±7.2	92.8±7.2	.001
HDL-C	49.4±0.1	52.2±0.1	53.5±1.5	46.4±0.8	46.6±0.4	44.9±1.2	45.3±1.9	< .001
Triglycerides	137.6±0.8	136.2±0.8	211.9±30.5	203.2±12.1	160.4±3.4	165.2±9.4	148.2±9.4	< .001
Fe (umol/dL)	117.5±0.6	118.0±0.6	114.2±9.4	124.4±7.3	101.5±1.8	91.30±5.6	73.36±5.1	< .001
Ferritin (ng/mL)	90.9±0.9	90.3±0.9	127.9±20.2	138.0±16.0	93.0±3.0	111.0±8.9	142.7±26.4	< .001
Hb (g/dL)	14.3±0.0	14.3±0.0	14.7±0.2	14.7±0.1	13.7±0.1	12.8±0.1	11.2±0.2	< .001
FBS (mg/dL)	97.6±0.2	97.1±0.1	120.9±5.7	123.7±3.2	108.2±1.0	111.2±3.3	114.6±6.3	< .001

***Note*:** Values are sample n, weighted mean ± SE or sample n, weighted percentage (SE) unless otherwise indicated; P-value by ANOVA or Chi-square test as appropriate. Abbreviations: DM = Diabetes mellitus; WC = Waist circumference; Cr = Creatinine; Total-C = Total Cholesterol; Hb = Hemoglobin; FBS = Fasting Blood Sugar.

### Factors associated with obesity

Table 4 presents the factors associated with general obesity after controlling for all potential confounding factors (sociodemographic, health-related factors, clinical factors and comorbidities) (Model 5). These factors were CKD stage 3a, CKD stage 3b, age, male sex, living with a spouse, unemployment, sleep duration, current smoking, EQ-5D, perceived stress, suicidal ideation, comorbidities (ischemic heart disease and HTN), SBP/DBP, total cholesterol, high-density lipoprotein (HDL) level, Hb level, and FBG. The likelihood of being obese was higher in participants with CKD stage 3a (adjusted OR 1.30, 95% CI = 1.09 to 1.55) or stage 3b (adjusted OR 1.91, 95% CI = 1.24 to 2.94), comorbidities (i.e., ischemic heart disease and HTN), or increased SBP, DBP, total cholesterol, Hb level, or FBG. In contrast, the likelihood of being obese was reduced by the following factors: older age, male sex, living without a spouse, unemployed status, current smoking status, longer sleep duration, and higher EQ-5D and HDL level (See Model 5 of Table 3 for adjusted OR and 95% CI).

**Table 4:** Factors associated with general obesity in different models (A-OR = Adjusted Odd Ratios).

	Model 1		Model 2		Model 3		Model 4		Model 5	
	OR	95% CI	OR	95% CI	OR	95% CI	OR	95% CI	OR	95% CI
Non-CKD	Reference		Reference		Reference		Reference		Reference	
CKD Stage 1	1.29	(0.80 to 2.07)	1.24	(0.73 to 2.09)	1.48	(0.85 to 2.61)	1.02	(0.55 to 1.90)	0.70	(0.34to 1.43)
CKD Stage 2	2.47	(1.79 to 3.40)	2.14	(1.53 to 2.99)	2.12	(1.50 to 3.00)	1.63	(1.14 to 2.34)	0.99	(0.66 to 1.47)
CKD Stage 3a	1.64	(1.43 to 1.90)	1.48	(1.28 to 1.71)	1.45	(1.24 to 1.69)	1.21	(1.02 to 1.42)	1.30	(1.09 to 1.55)
CKD Stage 3b	1.77	(1.28 to 2.45)	1.66	(1.17 to 2.36)	1.76	(1.21 to 2.55)	1.60	(1.07 to 2.38)	1.91	(1.24 to 2.94)
CKD Stage 4/5	1.29	(0.71 to 2.35)	1.32	(0.69 to 2.52)	1.57	(0.79 to 3.14)	1.01	(0.47 to 2.15)	1.05	(0.43 to 2.55)
Age, per 10-y older			1.04	(1.01 to 1.06)	1.02	(0.99 to 1.04)	0.89	(0.87 to 0.92)	0.84	(0.81 to 0.87)
Male, sex			1.50	(1.42 to 1.59)	1.48	(1.38 to 1.60)	1.33	(1.23 to 1.43)	0.68	(0.62 to 0.76)
Elementary to middle school			1.26	(1.17 to 1.36)	1.21	(1.12 to 1.32)	1.16	(1.06 to 1.26)	1.13	(1.03 to 1.24)
Residential area, rural			1.05	(0.97 to 1.13)	1.04	(0.95 to 1.13)	1.07	(0.98 to 1.16)	1.07	(0.98 to 1.17)
Living without spouse			0.81	(0.75 to 0.87)	0.79	(0.73 to 0.85)	0.74	(0.69 to 0.80)	0.82	(0.76 to 0.89)
Currently Unemployed			0.86	(0.81 to 0.92)	0.82	(0.77 to 0.88)	0.81	(0.76 to 0.87)	0.84	(0.78 to 0.91)
***Health-related Factors***										
Sleep Duration (hr.)					0.97	(0.94 to 0.99)	0.96	(0.94 to 0.99)	0.95	(0.93 to 0.97)
Current smoking					1.00	(0.92 to 1.08)	1.02	(0.94 to 1.10)	0.88	(0.81 to 0.96)
Alcohol drinking					0.98	(0.92 to 1.05)	0.92	(0.86 to 0.98)	1.05	(0.98 to 1.13)
Perceived health status										
Very good/Good					Reference		Reference		Reference	
Fair					1.13	(1.06 to 1.20)	1.07	(1.00 to 1.14)	1.00	(0.93 to1.08)
Poor/Very poor					1.19	(1.08 to 1.31)	1.04	(0.94 to 1.15)	1.01	(0.91 to 1.12)
EQ-5D					0.45	(0.33 to 0.60)	0.40	(0.30 to 0.55)	0.40	(0.29 to 0.55)
Depressive Symptom (Yes)					0.92	(0.84 to 1.02)	0.94	(0.85 to 1.04)	0.94	(0.84 to 1.05)
Perceived psychological stress					1.15	(1.07 to 1.24)	1.13	(1.05 to 1.22)	1.11	(1.02 to 1.20)
Suicidal ideation					0.90	(0.82 to 0.99)	0.90	(0.81 to 0.99)	0.89	(0.80 to 0.99)
***Comorbidity***										
Cancer							0.85	(0.70 to 1.03)	0.90	(0.74 to 1.10)
Ischemic heart disease							1.00	(0.81 to 1.24)	1.34	(1.08 to 1.65)
Stroke							0.94	(0.76 to 1.17)	1.11	(0.89 to 1.39)
Hypertension							2.59	(2.40 to 2.79)	1.66	(1.51 to 1.83)
Diabetes mellitus							1.61	(1.46 to 1.78)	1.08	(0.93 to 1.25)
Anemia							0.63	(0.56 to 0.71)	1.15	(1.00 to 1.34)
COPD or Asthma							0.98	(0.82 to 1.17)	1.09	(0.90 to 1.32)
***Examination/Lab Tests***										
SBP, per 10 mmHg									1.05	(1.02 to 1.09)
DBP, per 10 mmHg									1.27	(1.21 to 1.34)
Total cholesterol, per 10 mg/dL									1.10	(1.08 to 1.11)
HDL-C, per 10 mg/dL									0.63	(0.61 to 0.65)
Triglycerides, per 10 mg/dL									1.00	(1.00 to 1.01)
Hemoglobin per 1g/dL									1.17	(1.13 to 1.22)
Fasting blood sugar, per 10 mg/dL									1.08	(1.06 to 1.11)

***Note*:** 1) Model 1 was unadjusted; Model 2 was adjusted for the variables in Model 1 and sociodemographic factors (age, sex, education, residence, living with a spouse, and currently unemployed); Model 3 was adjusted for the variables in Model 2 and health-related characteristics (sleep duration, smoking, alcohol drinking, perceived health status, EQ-5D, depressive symptoms, perceived psychological stress, and suicidal ideation); Model 4 was adjusted for the variables in Model 3 and comorbidities (cancer, ischemic heart disease, stroke, hypertension, diabetes mellitus, anemia, and chronic obstructive pulmonary disease or asthma); Model 5 was adjusted for the variables in Model 4 and laboratory measurements (SBP, DBP, total cholesterol, high-density lipoprotein cholesterol, triglycerides, hemoglobin, and fasting blood sugar); 2) EuroQoL: visual analogue scale and level were examined only in 2010–2012; Fe levels were measured only in 2008–2012 and were therefore omitted from adjustments.

### Factors associated with abdominal obesity

Table 5 presents the factors associated with abdominal obesity. They were CKD stage 1, CKD stage 3a, male gender, education level (elementary to middle school), living in a rural area, sleep duration, alcohol drinking, perceived health status, EQ-5D, perceived stress, comorbidities (ischemic heart disease, HTN, and DM), SBP, DBP, total cholesterol, HDL level, Hb level and FBG after controlling for all potential confounding factors (Model 5). The likelihood of having abdominal obesity was higher in participants with stage 3a CKD than in participants without CKD (adjusted OR 1.23, 95% CI = 1.02 to 1.49), with lower education, perceived fair and poor/very poor health status, comorbidities (i.e., ischemic heart disease, HTN, and DM), increased SBP, DBP, total cholesterol, Hb level, or FBG, or who lived in a rural area. In contrast, the likelihood of abdominal obesity was lower in participants with the following factors: CKD stage 1, male sex, longer sleep duration, and higher EQ-5D and HDL level (See Model 5 of Table 4 for adjusted OR and 95% CI).

**Table 5:** Factors associated with abdominal obesity in different models (A-OR = Adjusted Odd Ratios).

	Model 1		Model 2		Model 3		Model 4		Model 5	
	OR	95% CI	OR	95% CI	OR	95% CI	OR	95% CI	OR	95% CI
Non-CKD	Reference		Reference		Reference		Reference		Reference	
CKD Stage 1	1.39	(0.86 to 2.24)	1.29	(0.76to 2.18)	1.25	(0.71 to 2.18)	0.78	(0.44 to 1.41)	0.52	(0.27 to 0.99)
CKD Stage 2	2.71	(1.97 to 3.72)	2.21	(1.59 to 3.08)	2.16	(1.52 to 3.07)	1.66	(1.14 to 2.41)	1.06	(0.73 to 1.55)
CKD Stage 3a	2.24	(1.95 to 2.57)	1.36	(1.17 to 1.58)	1.40	(1.19 to 1.65)	1.18	(0.98 to 1.40)	1.23	(1.02 to 1.49)
CKD Stage 3b	2.20	(1.58 to 3.08)	1.21	(0.84 to 1.72)	1.17	(0.79 to 1.72)	1.07	(0.69 to 1.65)	1.22	(0.75 to 1.96)
CKD Stage 4/5	1.45	(0.82 to 2.56)	1.01	(0.56 to 1.84)	1.14	(0.59 to 2.18)	0.92	(0.45 to 1.88)	0.99	(0.43 to 2.30)
Age, per 10-y older			1.21	(1.17 to 1.24)	1.20	(1.17 to 1.24)	1.07	(1.03 to 1.11)	1.03	(0.99 to 1.07)
Male, sex			1.22	(1.15 to 1.30)	1.15	(1.06 to 1.24)	0.99	(0.91 to 1.08)	0.51	(0.46 to 0.57)
Elementary to middle school			1.36	(1.26 to 1.48)	1.28	(1.17 to 1.40)	1.19	(1.09 to 1.31)	1.16	(1.05 to 1.28)
Residential area, rural			1.16	(1.05 to 1.27)	1.15	(1.04 to 1.27)	1.19	(1.08 to 1.33)	1.20	(1.08 to 1.34)
Living without spouse			0.92	(0.86 to 0.99)	0.90	(0.83 to 0.97)	0.86	(0.79 to 0.93)	0.92	(0.84 to 1.01)
Currently Unemployed			0.99	(0.93 to 1.06)	0.95	(0.88 to 1.03)	0.94	(0.87 to 1.02)	0.98	(0.91 to 1.07)
***Health-related Factors***										
Sleep Duration (hr.)					0.98	(0.96 to 1.01)	0.98	(0.96 to 1.01)	0.97	(0.94 to 1.00)
Current smoking					1.10	(1.01 to 1.20)	1.12	(1.03 to 1.23)	0.98	(0.89 to 1.07)
Alcohol drinking					1.06	(0.98 to 1.13)	1.00	(0.93 to 1.08)	1.15	(1.07 to 1.25)
Perceived health status										
Very good/Good					Reference		Reference		Reference	
Fair					1.22	(1.13 to 1.32)	1.15	(1.07 to 1.25)	1.10	(1.01 to 1.19)
Poor/Very poor					1.42	(1.28 to 1.58)	1.24	(1.11 to 1.38)	1.23	(1.10 to 1.38)
EQ-5D					0.46	(0.34 to 0.63)	0.39	(0.28 to 0.54)	0.39	(0.28 to 0.55)
Depressive Symptom (Yes)					0.89	(0.80 to 0.99)	0.90	(0.80 to 1.01)	0.91	(0.80 to 1.02)
Perceived psychological stress					1.18	(1.09 to 1.28)	1.17	(1.07 to 1.27)	1.14	(1.05 to 1.25)
Suicidal ideation					1.00	(0.89 to 1.11)	1.00	(0.89 to 1.12)	1.01	(0.90 to 1.13)
***Comorbidity***										
Cancer							0.84	(0.69 to 1.01)	0.89	(0.73 to 1.07)
Ischemic heart disease							1.04	(0.85 to 1.27)	1.32	(1.06 to 1.64)
Stroke							0.86	(0.68 to 1.07)	0.97	(0.76 to 1.22)
Hypertension							2.37	(2.19 to 2.57)	1.61	(1.46 to 1.78)
Diabetes mellitus							1.85	(1.68 to 2.05)	1.22	(1.05 to 1.43)
Anemia							0.50	(0.44 to 0.57)	0.94	(0.80 to 1.11)
COPD or Asthma							1.03	(0.86 to 1.25)	1.13	(0.92 to 1.38)
***Examination/Lab Tests***										
SBP, per 10 mmHg									1.07	(1.03 to 1.10)
DBP, per 10 mmHg									1.16	(1.11 to 1.22)
Total cholesterol, per 10 mg/dL									1.08	(1.07 to 1.09)
HDL-C, per 10 mg/dL									0.65	(0.63 to 0.68)
Triglycerides, per 10 mg/dL									1.00	(1.00 to 1.01)
Hemoglobin (g/dL)									1.21	(1.16 to 1.26)
Fasting blood sugar, per 10 mg/dL									1.08	(1.06 to 1.11)

***Note*:** 1) Model 1 was unadjusted; Model 2 was adjusted for the variables in Model 1 and sociodemographic factors (age, sex, education, residence, living with a spouse, and currently unemployed); Model 3 was adjusted for the variables in Model 2 and health-related characteristics (sleep duration, smoking, alcohol drinking, perceived health status, EQ-5D, depressive symptoms, perceived psychological stress, and suicidal ideation); Model 4 was adjusted for the variables in Model 3 and comorbidities (cancer, ischemic heart disease, stroke, hypertension, diabetes mellitus, anemia, and chronic obstructive pulmonary disease or asthma); Model 5 was adjusted for the variables in Model 4 and laboratory measurements (SBP, DBP, total cholesterol, high-density lipoprotein cholesterol, triglycerides, hemoglobin, and fasting blood sugar); 2) EuroQoL: visual analogue scale and level were examined only in 2010–2012; Fe levels were measured only in 2008–2012 and were therefore omitted from adjustments.

## Discussion

It is thought that obese people are more likely to develop CKD and ESKD because of hemodynamic and histopathological changes in the kidney in the setting of obesity [4, 19, 20]. In this study, we hypothesized that if obesity is an important contributor to the development and progression of CKD, the prevalence of obesity should become higher as CKD progresses. Furthermore, we assumed that if there is a significant relationship between CKD and obesity, CKD itself might be a contributing factor to obesity. If this is the case, then patients will be in a vicious cycle if they have both CKD and obesity. To our knowledge, this is the first study to explore the relationship between obesity/abdominal obesity and CKD according to the different stages of CKD.

The findings of the present study can be summarized as follows: (1) general obesity and abdominal obesity were more prevalent in CKD groups compared to the non-CKD group; (2) the prevalence of general obesity and abdominal obesity was highest in stage 2 CKD; (3) stages 3a/3b CKD were the significant factors associated with general obesity, and stage 3a CKD was significantly associated with abdominal obesity; and (4) the presence of various comorbidities was an independent contributor to the development of both general obesity and abdominal obesity.

Our study showed that people with CKD stages 3a and 3b were more likely to have general obesity and people with CKD stages 3a were more likely to have abdominal obesity. It has been suggested that WC, not BMI, might be a better indicator of obesity-related health risk [15]. Adiposity resulting from abdominal obesity has been suggested to play a direct role in causing or worsening CKD by inducing endocrine dysfunction or chronic inflammation [21, 22]. The known deleterious role of abdominal adiposity in CKD and the findings of this study could suggest that CKD stage 3a might be the most significant factor associated with both general obesity and abdominal obesity. Because numerous population-based studies have established a significant association between obesity and the development and progression of CKD [3–9], our study findings might simply substantiate previous findings. However, our study showed for the first time that early-stage CKD could be a factor contributing to obesity, unlike previous studies that demonstrated the role of obesity in CKD pathogenesis. Although these findings are interesting, we can only speculate why early stages of CKD could contribute to obesity. In most cases, CKD develops secondary to cardiovascular disease or type 2 diabetes, among other diseases. Thus, the development of CKD adds to patient comorbidity, and may influence the quality of life. This may lead to adoption of a more sedentary lifestyle with less exercise, which contributes to weight gain in such patients. Alternatively, the levels of peptides in blood that regulate energy expenditure, such as *ghrelin or leptin*, could be different according to the CKD stage, as both ghrelin and leptin are metabolized in the kidney, leading to a skewed indication of positive energy balance in patients with early CKD [23, 24]. At this point, we are unsure whether early CKD can be a risk factor for development of obesity because our observation could be a simple association between early CKD and obesity. Thus, further research is needed to clarify our study findings.

The present study also found a relatively lower prevalence of general obesity and abdominal obesity in CKD stage 4/5 than other CKD stages (Table 1), and there was no association between general obesity or abdominal obesity and CKD stage 4/5 (Tables 4 and 5). These findings could be due to either more dietary restriction or more severe nausea, leading to poor appetite in people with CKD stage 4/5. Interestingly, obesity has been associated consistently with lower mortality rates in patients with advanced CKD and ESKD [7, 10–12].

Viewed in combination, these previous findings and those of the current study may suggest that obesity is strongly associated with the development and progression of CKD at an early stage but confer a benefit in advanced CKD. In other words, obesity might be a double-edged sword for CKD patients. Thus, a therapeutic approach to overweight and obesity in patients with advanced CKD should be approached carefully.

Regarding the factors associated with general obesity /abdominal obesity other than CKD (Tables 4 and 5), it is notable that male gender was negatively associated with both general obesity and abdominal obesity in this study. Although controversial, no obvious gender differences in obesity prevalence have been detected to date [25, 26]. Furthermore, older age and unemployment were negatively associated with obesity in the present study. It has been reported that the prevalence of obesity is highest in middle-aged adults (40–59 years) and lowest in young adults (20–39 years) [27]. Regarding comorbidity factors, HTN was significantly associated with both general obesity and abdominal obesity and DM was only associated with abdominal obesity. Given that obesity is a well-established major risk factor for cardiovascular diseases and type-II diabetes, this is not a surprising finding [2]. Further, our study findings also agree that WC, not BMI, might be a better indicator of obesity-related health risk [15, 22].

Of note, we have used the Korean definition in this study to define general and abdominal obesity [14]. A recent study has reported that the percentage of body fat is higher in Asians than that observed in the white population at the same body mass index levels [14]. Therefore, cutoff values to define an overweight status and obesity should be lower in Asian populations, and these cutoff values should be based on morbidity and mortality associated with obesity [28]. This is why we have used the Korean definition of obesity.

The findings of the present study have a few important clinical implications. First, these findings are congruent with those of previous studies in that they revealed a significant association between general obesity/abdominal obesity and CKD. Second, we further observed that the association between general obesity/abdominal obesity and CKD disappears when CKD reaches an advanced stage (stage 4/5). This means that weight loss might be a sign of poor clinical outcomes in patients with severe CKD. Third, CKD itself could be a contributing factor to obesity in moderate CKD (stage 3). Given the role of obesity in the progression of CKD, patients with moderate CKD can enter a vicious cycle if they have both CKD and obesity. In conclusion, the findings of this study clearly support the idea that weight loss is a good potential intervention for the prevention of progression in moderate CKD (stage 3), but not severe CKD (stage 4/5).

The main strength of this study is that it was done in a large number of individuals using nationwide and representative survey data. Also the ethnic homogeneity of study participants can be another strength since it enabled us to reduce the potential confounding effects of different races and ethnicities to address the relationship between obesity and CKD. However, this ethnic homogeneity of study participant could be viewed as limitation since further studies will be needed in populations with different ethnic backgrounds before generalization of our study findings. Furthermore, as mentioned earlier, the cross-sectional design of the KNHANES precluded the identification of a causal relationship, and this represents another limitation of the present study. Due to the nature of cross-sectional studies, patients with acute kidney injury could have been accidentally included in this study given that a single measurement of eGFR might not represent CKD accurately.

### Conclusions

In conclusion, there is increased prevalence of obesity in patients with CKD in South Korea except for severe CKD patients. Stages 3 CKD seems to be an independent factor for obesity as well. The findings of this study clearly support the idea that weight loss might be a good potential intervention for the prevention of disease progression in moderate CKD (stage 3), but not severe CKD (stage 4/5).
